# Co-Infections and Superinfections in COVID-19 Critically Ill Patients Are Associated with CT Imaging Abnormalities and the Worst Outcomes

**DOI:** 10.3390/diagnostics12071617

**Published:** 2022-07-03

**Authors:** Nicolò Brandi, Federica Ciccarese, Caterina Balacchi, Maria Rita Rimondi, Cecilia Modolon, Camilla Sportoletti, Chiara Capozzi, Matteo Renzulli, Alexandro Paccapelo, Andrea Castelli, Rita Golfieri

**Affiliations:** 1 Department of Radiology, IRCCS Azienda Ospedaliero-Universitaria di Bologna, Via Albertoni 15, 40138 Bologna, Italy; ciccarese.f@gmail.com (F.C.); caterinabalacchi@libero.it (C.B.); matteo.renzulli@aosp.bo.it (M.R.); alexandro.paccapelo@aosp.bo.it (A.P.); rita.golfieri@unibo.it (R.G.); 2 Cardio-Thoracic Radiology Unit, University Hospital S.Orsola-Malpighi, 40138 Bologna, Italy; mariarita.rimondi@aosp.bo.it (M.R.R.); cecilia.modolon@aosp.bo.it (C.M.); csportoletti@hotmail.com (C.S.); 3 Cardio-Anesthesiology Unit, Cardio-Thoracic-Vascular Department, S.Orsola Hospital, University of Bologna, 40138 Bologna, Italy; capozzi.chiara@gmail.com (C.C.); andrea.castelli@aosp.bo.it (A.C.)

**Keywords:** COVID-19, ARDS, lung CT, intensive care, superinfection, coinfection, bacterial infections, fungal infections, cavitation, bronchiectasis, consolidation

## Abstract

Background: Bacterial and fungal co-infections and superinfections have a critical role in the outcome of the COVID-19 patients admitted to the Intensive Care Unit (ICU). Methods: The present study is a retrospective analysis of 95 patients admitted to the ICU for COVID-19-related ARDS during the first (February–May 2020) and second waves of the pandemic (October 2020–January 2021). Demographic and clinical data, CT imaging features, and pulmonary and extra-pulmonary complications were recorded, as well as the temporal evolution of CT findings when more than one scan was available. The presence of co-infections and superinfections was registered, reporting the culprit pathogens and the specimen type for culture. A comparison between patients with and without bacterial and/or co-infections/superinfections was performed. Results: Sixty-three patients (66.3%) developed at least one confirmed co-infection/superinfection, with 52 (82.5%) developing pneumonia and 43 (68.3%) bloodstream infection. Gram-negative bacteria were the most common co-pathogens identified and *Aspergillus* spp. was the most frequent pulmonary microorganism. Consolidations, cavitations, and bronchiectasis were significantly associated with the presence of co-infections/superinfections (*p* = 0.009, *p* = 0.010 and *p* = 0.009, respectively); when considering only patients with pulmonary co-pathogens, only consolidations remained statistically significative (*p* = 0.004). Invasive pulmonary aspergillosis was significantly associated with the presence of cavitations and bronchiectasis (*p* < 0.001). Patients with co-infections/superinfections presented a significantly higher mortality rate compared to patients with COVID-19 only (52.4% vs. 25%, *p* = 0.016). Conclusions: Bacterial and fungal co-infections and superinfections are frequent in COVID-19 patients admitted to ICU and are associated with worse outcomes. Imaging plays an important role in monitoring critically ill COVID-19 patients and may help detect these complications, suggesting further laboratory investigations.

## 1. Introduction

Although it has been more than 2 years since the first outbreak, the coronavirus disease 2019 (COVID-19) pandemic is still having a profound and devastating impact on global healthcare systems. COVID-19 is responsible for a respiratory disease whose broad spectrum of severity ranges from asymptomatic or mildly symptomatic infection to severe bilateral pneumonia, which may lead to acute respiratory distress syndrome (ARDS), requiring non-invasive or invasive mechanical ventilation and Intensive Care Unit (ICU) admission [1].

Several complications can arise during ICU stay, from both COVID-19 extensive lung damage and extra-pulmonary involvement, as well as those secondary to mechanical supporting systems [2,3,4,5,6]. Among these, bacterial and fungal co-infections and superinfections (or secondary infections) play an important role in COVID-19 disease and have been associated with increased morbidity and mortality, especially in critically ill patients [7,8,9,10,11].

Co-infections, defined as infections that occur ≤48–72 h after hospital admission, are rare in COVID-19 patients and are reported in about 7% of hospitalized patients [12,13]. On the contrary, secondary infections (or superinfections), defined as infections that emerge during the course of the illness or hospital stay (i.e., >48–72 h after admission), are more frequently diagnosed, especially in critically ill patients [13,14]. In particular, the incidence of secondary pulmonary infections in hospitalized patients was reported to be 16% (4.8–42.8%) and 6.3% (0.9–33.3%) for bacterial and fungal infections, respectively [10]. In critically ill patients, the incidence of superinfections is much higher, reaching up to 45% of cases [15,16,17,18], and is constituted mostly of pneumonia (50%) and bloodstream infections (34%) [19].

The reported higher frequency of co-pathogen infections in COVID-19 patients admitted to ICU has been associated with various risk factors. In fact, in addition to the diffuse alveolar damage and the COVID-19-induced immunosuppressed state [20], ICU patients undergo several invasive procedures (such as endotracheal tube and venous catheter placement), are generally weaker due to the more severe form of illness, have more underlying comorbidities, and are treated longer with anti-inflammatory and antibiotic therapies, thus, resulting in extreme vulnerability and greater susceptibility to both bacterial and fungal infections [16,21].

Focusing on microbiological etiology, the majority of COVID-19 patients (about 50–70%) is affected by simultaneous bacterial and fungal infections and mixed infections (with more than one bacterium isolated from the same site), seen in about 25% of cases [7,22].

According to culture results, the most common co-pathogens identified were mainly Gram-negative bacteria, including *Klebsiella pneumoniae, Pseudomonas aeruginosa*, *Haemophilus influenzae*, *Escherichia Coli,* and *Acinetobacter baumannii*, although *Mycoplasma pneumoniae* and *Staphylococcus aureus* have also been frequently observed [10,14,16,23]. Among the etiologic agents responsible for fungal co-infections and superinfections, *Aspergillus* spp. and *Candida* spp. were the most frequently detected species [24]; however, less frequent opportunistic fungal pathogens are increasingly reported, including *Mucorales*, *Histoplasma* spp., *Cryptococcus* spp., and *Pneumocystis jirovecii*.

Distinguishing severe viral pneumonia from bacterial and fungal co-infection/superinfection is challenging and clinicians should always rely on a combination of the clinical course of the disease and the results of both laboratory tests and imaging.

During the early phases of the pandemic, when viral tests were not available or were scarce, imaging provided considerable help in the diagnosis of COVID-19 pneumonia. At present, following the advancement in diagnostic laboratoristic techniques, its role has evolved and now imaging is pivotal in the detection and monitoring of COVID-19 complications, including co-infections and superinfections [25]. In fact, a sudden increase in X-ray opacities and/or the presence of atypical features for COVID-19 (such as lobar consolidation, pleural effusion, mediastinal lymphadenopathy, and cavitation) is strongly suspected of bacterial superimposed infection, especially if paired with rapid clinical deterioration of the patient [26]. Moreover, semi-automatic segmentation of CT images has recently been suggested as a tool for the extraction of radiomic features that may predict several clinical endpoints of COVID-19 patients, including ICU admission and the need for ventilators [27].

Several studies reported a huge discrepancy between empiric antimicrobial prescribing (72–100%) and the reported incidence of secondary infection (8–15%) [7,15]. Therefore, in the context of rising levels of antimicrobial resistance and approximately one-third of all the infectious episodes being due to multi-drug-resistant organisms (35%), understanding the proportion of COVID-19 patients with co-infection and secondary infection and the culprit pathogens is crucial for a correct treatment of these patients and to ensure responsible use of antibiotics [19].

The first aim of the present study was to report the prevalence of bacterial and fungal co-infections and superinfections associated with COVID-19 pneumonia in ICU patients. The second aim was to evaluate the main chest CT imaging features of COVID-19 patients with co-infections/superinfections and to compare them with those of patients with COVID-19-only pneumonia, to identify those which could help in the correct identification and prompt diagnosis of superimposed infections. Finally, clinical outcomes of COVID-19 patients with co-infections/superinfections were analyzed, with the aim to determine the burden of these complications on COVID-19 critically ill patients.

## 2. Materials and Methods

### 2.1. Patient Population and Study Design

This study was an observational, retrospective, single-center study and was approved by the local institution review board (IRB). Informed consent was waived by the IRB due to the retrospective nature of the study.

**Figure 1:**
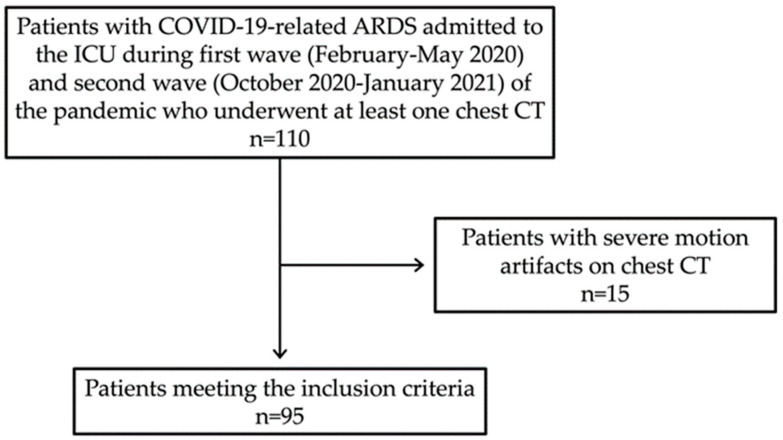
Flow diagram of patient selection in the study.

We consecutively enrolled all the patients admitted to the ICU for COVID-19 pneumonia from February to May 2020 (first wave) and from October 2020 to January 2021 (second wave), which represented the two peaks of infection in our country with the majority of hospital and ICU beds dedicated almost exclusively to COVID-19 patients. To be included in the study, all the patients had to receive a diagnosis of COVID-19-related ARDS and undergo at least one chest CT at our Institute. In particular, COVID-19 ARDS was diagnosed when a patient with confirmed COVID-19 infection developed acute respiratory distress meeting the Berlin 2012 ARDS diagnostic criteria, which include (a) acute hypoxemic respiratory failure (b) developing within 1 week of onset of symptoms (c) with bilateral air space opacities on X-ray or CT (d) not explained by cardiac failure or fluid overload [28,29]. Patients with COVID-19-related ARDS investigated only with other imaging techniques (X-ray or Ultrasound) and/or with CT performed outside our Institute were excluded. Finally, all the patients with severe motion artifacts on chest CT (*n* = 15) were excluded from the study (Figure 1).

The following clinical data were collected from patients’ discharge letters: age, gender, body mass index (BMI), time of onset of the disease and clinical outcome (exitus during ICU stay or recovery), white blood cell, and lymphocyte counts at the time of admission to ICU. In addition, the presence of co-infections (≤48–72 h after admission) and superinfections (>48–72 h after admission) was registered, making sure to report the responsible bacterial and/or fungal pathogens and the specimen type for culture (endotracheal aspirate/bronchoalveolar lavage, blood, and urine). All samples were processed according to internal standards to allow the detection of conventional bacteria and slow-growing pathogens, such as mycobacteria and fungi. In addition, all respiratory and blood samples underwent galactomannan test for the detection of mold infections. According to the literature, ref. [30] the definition of invasive pulmonary aspergillosis was modified from the AspICU algorithm [31] and was based on the concomitant presence of clinical, radiological, and mycological criteria. Bloodstream infections were defined as a single positive blood culture for a likely pathogen or two or more positive blood cultures for common skin colonizers, without a concomitant microbiologically documented lower respiratory tract infection due to the same pathogen. We excluded from the study 40 cases of positive cultures (27 blood cultures, 9 urine cultures, and 4 tracheal aspirates) that were considered contaminations.

The treatment protocol consisted of the combined administration of azithromycin, hydroxychloroquine, steroids, tocilizumab, and low-molecular-weight heparin (antithrombotic prophylaxis), together with antibiotics in those cases where a bacterial co-pathogen was isolated.

### 2.2. CT Acquisition Technique and Image Analysis

Chest CT acquisitions were obtained with the patients in a supine position on a CT scanner dedicated only to patients affected by COVID-19 (64 slices GE light-speed VCT), with the following technical parameters: tube voltage: 120 kV, tube current modulation: 80–550 mAs, spiral pitch factor: 0.984, and collimation width: 26 × 0.6; reconstructions were obtained at a slice thickness of 1.25 × 1 mm.

Additional scans were performed for suspected extra-pulmonary complications: non-enhanced CT of the head in 34 patients, CT of the abdomen with contrast media injection in 36 patients, and CT angiography for the pulmonary vessels in 58 patients.

All the CT examinations were reviewed by accessing the PACS (Picture Archiving and Communication System) of our hospital by the team of expert radiologists involved in the study with a consensus reading [32,33]. In particular, the presence of ground-glass, crazy paving, consolidations, pleural effusion, pericardial effusion, cavitations, bronchiectasis, pneumothorax and pneumomediastinum, bronchial distortion, and reticulations was collected. Moreover, complications involving other organs were recorded, including acute pulmonary embolism, other thrombotic, or hemorrhagic complications, and abdominal and brain complications.

### 2.3. Statistical Analysis

Data are presented as means ± standard deviations and frequencies. The Fisher’s exact test, Student’s T test, and Z test for two proportions were used.

Two-tailed *p*-values < 0.05 were considered statistically significant. Statistical analysis was carried out using IBM SPSS Statistics for Windows, Version 25.0 (Armonk, NY, USA: IBM Corp).

## 3. Results

During the study period, in total, 95 patients with COVID-19-related ARDS were admitted to the ICU to undergo at least one chest CT. In particular, 63 patients (66.3%) developed at least one confirmed nosocomial infection during hospitalization in the ICU wards, whereas 32 patients (33.7%) were affected by COVID-19 only. Only 6 patients (6/63, 9.5%; 6/95, 6.3%) developed a co-infection (≤48–72 h from admission) during hospitalization in the ICU, whereas the majority presented with superinfections (57/63, 90.5%, 57/95, 60%). The time taken for secondary pulmonary infection diagnosis was highly variable, between 3 and 23 days (mean 11 days) from ICU admission.

The majority of ICU patients were males (74.7%) and overweight (mean BMI: 29.53). No significant difference was noted regarding age, gender, and BMI between the two groups. Moreover, both groups did not differ regarding the white blood cell and lymphocyte counts at the time of admission to ICU, as well as for the presence of diabetes or other risk factors (including recent kidney transplant and lymphoproliferative disorders) (Table 1).

Of note, no differences were found between the first and the second waves, except for a higher BMI in patients admitted during the second wave (*p* = 0.007) (data not shown).

### 3.1. Prevalence of Bacterial and Fungal Superinfections and Co-Infections in COVID-19 Patients in ICU

Of the 63 patients with confirmed co-infection/superinfection, 33 (52.4%) had only bacterial infections, 27 (42.9%) were positive for both bacterial and fungal infections, and 3 (4.8%) had only fungal infections. It should be noted that most patients develop more than one infection during hospitalization in ICU wards (73%). During the study period, 162 microbes were identified, with mixed infection (more than one bacterium or fungus isolated from the same site) in 36.5% (23/63) of patients. The microbiology of the infections is described in Table 2.

According to culture and laboratory results, the most common bacterial co-pathogens identified were mainly Gram-negative (66.9%), which were identified in 53 patients (84.1%), whereas Gram-positive bacteria were isolated only in 35 patients (55.5%).

Fifty-two (52/63, 82.5%; 52/95, 54.7%) ICU patients with COVID-19 pneumonia were identified to have a respiratory bacterial and/or fungal co-infection/superinfection in at least one of the sequential study periods. In particular, the most commonly detected pathogens were *Aspergillus* spp. (*n* = 19) and *Klebsiella pneumoniae* (*n* = 16), followed by *Pseudomonas aeruginosa* (*n* = 8), *Acinetobacter baumannii* (*n* = 8), and *Staphylococcus aureus* (*n* = 7) and *Candida* spp. (*n* = 6).

Forty-three patients (43/63, 68.2%; 43/95, 45.3%) developed at least one bloodstream infection. Among the pathogens responsible for bloodstream infections, *Enterococcus faecium* (*n* = 11) was the most frequently observed, followed by *Acinetobacter baumannii* (*n* = 9), *Pseudomonas aeruginosa* (*n* = 8)*, Klebsiella Pneumoniae* (*n* = 8), and *Staphylococcus epidermidis* (*n* = 8).

Only 16 patients (16/63, 25.4%, 16/95, 16.8%) developed a urine infection. According to urine culture results, the most common co-pathogens identified were Gram-negative bacteria, including *Acinetobacter baumannii* (*n* = 4), *Escherichia coli* (*n* = 3), and *Klebsiella pneumonia* (*n* = 2).

### 3.2. Chest CT Imaging Features Associated with Superinfections and Co-Infections

The CT scan was performed at an average of 21.2 ± 13.2 days from disease onset, while the follow-up scan was performed at 33.6 ± 16.2 days. The chest CT findings in the overall populations and in the two subgroups are reported in Table 1.

**Figure 2:**
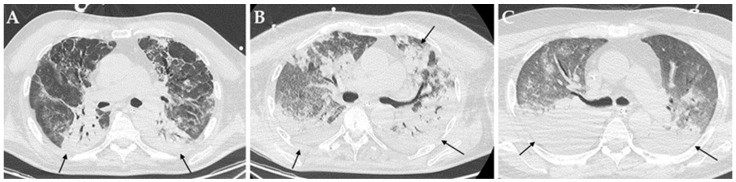
Axial HRCT images of different patients (**A**–**C**) with COVID-19 ARDS admitted to ICU showing bilateral consolidations (black arrows). In particular, all these patients developed a pulmonary superinfection by *Klebsiella pneumoniae* (**A**), *Pseudomonas aeruginosa* plus *Acinetobacter baumannii* (**B**) and *Staphylococcus aureus* plus *Candida albicans*.

**Figure 3:**
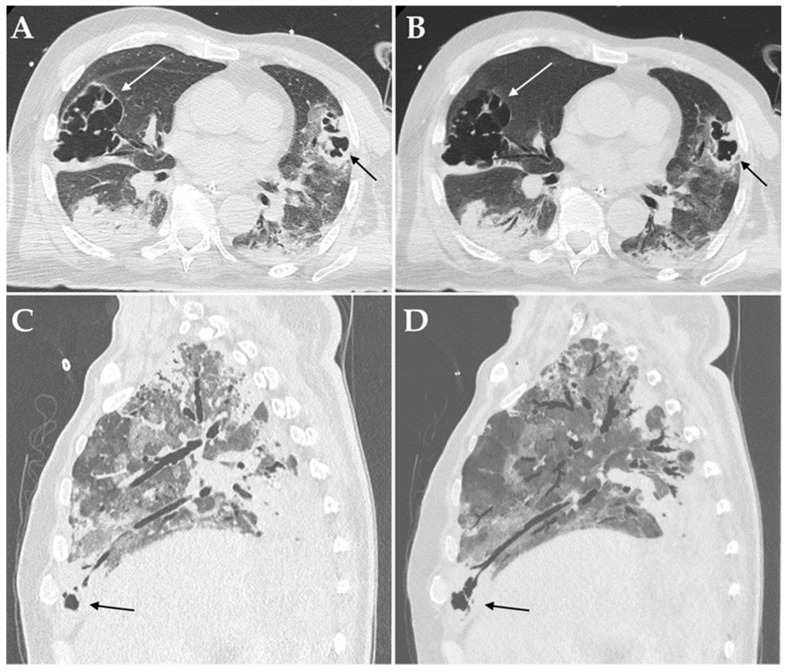
Axial HRCT images of a patient admitted to ICU with confirmed superinfection by *Aspergillus fumigatus* showing pulmonary consolidations in the lower lobes complicated by bilateral cavitations (white and black arrows in (**A**)); Minimum Intensity Projection (MIP) reconstruction of the same patient demonstrating communication between the cavitations and the bronchial tree (white and black arrows in (**B**)). Sagittal HRCT images of a patient admitted to ICU with confirmed superinfection by *Aspergillus niger* showing pulmonary consolidations in the anterior-basal segment of the right lower lobe complicated by small cavitations (black arrow in (**C**)); Minimum Intensity Projection (MIP) reconstruction of the same patient demonstrating communication between the cavitation and the bronchial tree (black arrows in (**D**)).

**Figure 4:**
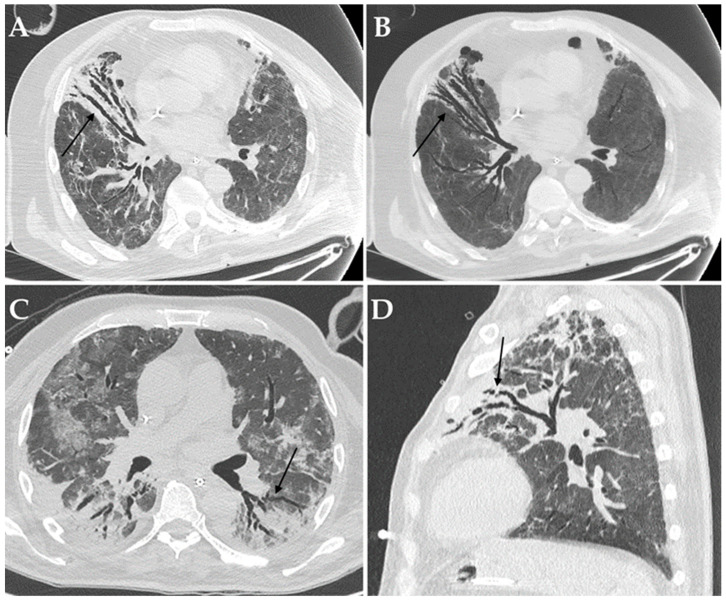
Axial HRCT images of a patient with COVID-19 ARDS admitted to ICU with bronchiectasis in the middle lobe (black arrow in (**A**)), being more evident at Minimum Intensity Projection (MIP) reconstruction (black arrow in (**B**)); *Aspergillus niger* was later detected in the bronchoalveolar lavage of the patient. Axial HRCT image of a different patient with COVID-19 ARDS with superinfection by *Klebsiella pneumoniae* and bronchiectasis in the dorsal segment of the left upper lobe (black arrow in (**C**)). Sagittal HRCT image of a different patient with COVID-19 ARDS showing bronchiectasis in the left upper lobe (black arrow in (**D**)) and presenting with elevated serum-galactomannan levels compatible with *Aspergillus* spp. superinfection.

Among imaging findings, consolidations (Figure 2), cavitations (Figure 3) and bronchiectasis (Figure 4) were statistically significantly associated with the presence of a bacterial and/or fungal co-infection/superinfection (*p* = 0.009, *p* = 0.010 and *p* = 0.009, respectively). When considering only patients with pulmonary co-pathogens, consolidations, cavitations, and bronchiectasis were more frequent in these patients compared to patients with co-infections/superinfections in other systems (100% vs. 72.7%, 30.8% vs. 0%, and 30.8% vs. 0%, respectively); however, only consolidations were statistically associated with the presence of a pulmonary bacterial and/or fungal co-pathogen (*p* = 0.004) (Table 3).

When considering the most common culprit pathogen (i.e., *Aspergillus* spp.), it emerged that invasive pulmonary aspergillosis was significantly associated with the presence of cavitations and bronchiectasis (*p* < 0.001); consolidations were present in all the cases of pulmonary co-infections/superinfections and, thus, did not generate any results.

Notably, the follow-up scan documented a reduction in ground-glass opacities (*p* = 0.036), while bronchial distortion and reticulations increased progressively over time (*p* = 0.011 and *p* = 0.001, respectively) (Table 4).

### 3.3. Clinical Outcomes of Superinfections and Co-Infections in COVID-19 Patients in ICU

The overall mortality rate of the entire ICU population was 43.2%. In particular, patients with co-infections/superinfections presented a significantly higher mortality rate compared to patients with COVID-19 only (52.4% vs. 25%, *p* = 0.016) (Table 1). No correlation was found regarding mortality for BMI, age, gender, or the development of other radiological complications.

Forty-one patients (43.2%) developed at least one adverse event detected by CT during hospitalization in the ICU ward, including abdominal complications (21/95, 22.1%), acute pulmonary embolism (16/95, 16.8%), barotraumas (14/95, 14.7%), and ischemic brain lesions (5/95, 5.3%); in particular, abdominal complications included five small bowel occlusions, six bowel ischemia, four pancreatitis, four spontaneous hematomas, and two infected fluid collections. The rate of adverse events, as well as the number, did not differ between the two groups (Table 1).

## 4. Discussion

The current knowledge of both co-infections and secondary infections in COVID-19 is continuously evolving but remains poorly understood. It is becoming apparent that superimposed bacterial and fungal infections are more frequent in critically ill COVID-19 patients and can be associated with worse outcomes. However, the prompt and correct detection of these dangerous complications is still challenging for both clinicians and radiologists; thus, further data are needed.

The present study reported a high rate of critically ill COVID-19 patients who developed at least one co-infection/superinfection during their ICU hospitalization (66.3%), which is high but similar to other reports [11,16,17]. However, based on the data in the literature, the percentage of COVID-19 patients with co-infections or secondary infections may be highly variable, ranging between 14% and 100%, depending on the differences in the inclusion criteria used, the specimen source, and/or population (both ICU and hospitalized COVID-19 patients vs. ICU patients only) [11,34,35]. The average time taken to diagnose secondary bacterial and fungal infections from ICU admission was 11 days (range: 3–23 days), which is very similar to literature reports [10].

In ICU, patients are extremely vulnerable and more susceptible to infection because of general weakness due to disease, weakening of defense mechanisms and length of hospitalization, catheter placement, and insertion of the endotracheal tube into artificial ventilation, urinary catheterization, and central vein [16]. In addition, in an effort to resolve underlying inflammation, COVID-19 infection triggers innate and adaptive immune responses, with the recruitment of macrophages, monocytes, T- and B-cells, and the release of cytokines [36]. This dysregulated immune response is seen to a greater degree in patients with severe COVID-19 infections and may lead to an immunosuppression stage following the proinflammatory phase [37]. Moreover, COVID-19 patients are more prone to receive corticosteroids and monoclonal antibodies compared to other patients; thus, the virus-induced immunosuppression effect could be further amplified [10,38].

As already reported in the literature [17], bacterial and/or fungal pneumonia represented the most common superimposed infections. In particular, the identification of a high percentage of Gram-negative microorganisms in our population (mostly constituted of *Klebsiella pneumoniae*, *Pseudomonas aeruginosa,* and *Acinetobacter baumannii*) is consistent with the previous data reported in the literature [10,16,39,40] and differs from those identified during both seasonal and pandemic influenza (i.e., *Streptococcus pneumoniae.* and *Staphylococcus aureus*) [41,42,43,44].

*Aspergillus* spp. has been previously identified as the most common fungal microorganism responsible for secondary pulmonary infections, both in hospitalized and ICU patients [10,17], and this result was confirmed also in the present study (20% of the overall ICU patients with COVID-19 ARDS). Due to inconsistent definitions and diagnostic criteria of invasive aspergillosis in non-neutropenic critically ill patients [45,46,47], the actual prevalence of this entity in the COVID-19 patients admitted to ICU is still unclear [17,48]. Similar to the present results, Koehler et al. found invasive pulmonary aspergillosis in 26.3% of critically ill patients with COVID-19 ARDS [49], whereas Van Arkel et al. achieved the diagnosis in 19.4% of cases [50]. On the contrary, Versyck et al. [51], reported a lower prevalence of invasive pulmonary aspergillosis in their cohort of ICU COVID-19 patients (3.7%). The relatively high percentage of invasive pulmonary aspergillosis in the present population could be related to the fact that most of the patients only had one mycological criterion for it, thus, increasing diagnostic sensitivity. Moreover, according to the newly proposed consensus criteria [52], “proven” invasive pulmonary aspergillosis requires histopathological or direct microscopic detection, which was not performed in the present study, in which, therefore, all the diagnoses would be “probable”. Despite these considerations, the detection of invasive aspergillosis in COVID-19 patients is certainly not rare; therefore, clinicians should be aware of this possibility, especially in those critically ill or immunocompromised patients, in which this complication may further aggravate their already impaired state.

In the present study, bloodstream infections were confirmed as the second most frequent infective complication, with a significant proportion being Gram-negative pathogens [21,53,54]. The elevated prevalence of *Enterococcus* spp. has been highlighted in other studies and is supposedly related to the use of cephalosporins as early empirical treatment [55,56]. On the contrary, the relatively high rate of *Staphylococcus* spp. may instead reflect the burden of catheter-associated infections, especially in the overcrowded or makeshift ICUs of the early stages of the pandemic [57].

High-resolution chest CT can show atypical imaging features suspicious of co-infections or superinfections in COVID-19 patients, thus, helping clinicians to achieve a prompt and correct diagnosis. Despite the presence of consolidations, cavitations, and bronchiectasis, especially when newly appeared, being suggestive of bacterial and/or fungal superimposed infections, they are not pathognomonic. However, chest CT plays a critical role in determining the appropriateness of various downstream diagnostic procedures in these patients, in order to confirm or exclude the diagnosis.

Consolidations were found to be statistically associated with the presence of a co-infection/superinfection in critically ill COVID-19 patients; in addition, they proved to be the only imaging feature specifically linked to the presence of one or more respiratory bacterial and/or fungal co-pathogen. However, consolidations did not prove to be associated with a specific bacterial or fungal pathogen, since 100% of the patients with a superimposed respiratory infection presented with this imaging feature at CT imaging.

Cavitation is uncommon for viral pneumonias, including those due to the other human coronaviruses [58]. Nonetheless, the development of pulmonary cavitation in patients with severe COVID-19 lung disease, especially in those admitted to ICU, is not rare, with a reported incidence ranging from 11 to 56% [55,59,60]. It is unclear whether the secondary bacterial infection or invasive fungal infection contribute to the development of the cavities, or the cavity is formed due to SARS-CoV-2 itself due to micro-infarcts; moreover, the relatively high rate of these atypical imaging findings in ICU patients could also be related to the extensive use of invasive mechanical systems, which may lead to barotraumas [56,61,62]. According to the present results, fungal co-infections and superinfections should always be considered in the presence of cavitary lesions, with prompt microbiological testing performed for definitive diagnosis [63,64]. In particular, *Aspergillus* spp. has been described as one of the most common causes of cavitation [55,59].

The presence or the de-novo appearance or the volumetric increase in bronchiectasis have been previously associated with fungal or mycobacterial colonization in COVID-19 patients [65,66,67,68] and were also confirmed in the present series. Bronchiectasis refers to a slight bronchiolar dilatation of distant small bronchioles. In patients with bronchiectasis, respiratory pathogens’ growth is enhanced by deteriorated mucociliary clearance, thick mucosity, and the capability to escape the immune system response. Moreover, long-lasting mucous content in the small bronchioles, together with the inflammatory process in the lung parenchyma, may be responsible for inflammatory damage and progressive enlargement of the bronchioles, in the end, leading to lung cavities [56], which would explain why both these imaging features resulted in an association with fungal superinfections. Therefore, the radiologic detection of de-novo appearance or the volumetric increase in bronchiectasis should be warning signs for radiologists and lead clinicians to investigate for fungal superinfections.

Finally, the present study demonstrated that ICU COVID-19 patients with co-infections/superinfections were more likely to die compared to ICU COVID-19 patients who did not have a superimposed infection. This result is concordant with previous studies of COVID-19 critically ill patients admitted to ICU, where secondary infections are associated with negative clinical outcome in up to 80% of cases [11]. Moreover, nosocomial-infection-causing organisms have been increasingly reported to be resistant to common antibiotics and therapies [69]; thus, their successful management and proper treatment represent key factors to reduce hospitalization costs [12].

As an accessory finding, the present study demonstrated a progressive reduction in the ground-glass opacities in survived patients over time, paired with an increased detection for interstitial abnormalities, namely bronchial distortion and reticulations (57.6% and 63.6% at 33.6 days from disease onset, respectively). These percentages seemed to be higher than those reported for other coronaviruses or H1N1 and are currently termed “long-COVID”. Despite these findings emerging as a key research topic and showing a significant correlation with disease severity, further investigations are required, due to both the heterogeneity of studies and the short follow-up [70,71].

The strengths of this study comprise the large number of patients included, as well as the clear, complete collection of radiological and microbiologic data. However, there are several limitations that need to be addressed. Due to its retrospective nature, the decision to obtain cross-sectional imaging was clinically guided and not protocolized and treatment protocols changed over time, as novel clinical evidence became available and the pandemic evolved. However, the two groups of patients considered (enrolled during the first and second wave) had comparable epidemiological and clinical features, with variants of the virus not being detected. Secondly, all the data derived from a pre-vaccine era; thus, it would be interesting to verify whether vaccines or new variants can be associated with changes in the microbiological epidemiology or in the prognosis of COVID-19 patients. Moreover, it is a single-center study from a Tertiary Care hospital in the epicenter of the COVID-19 pandemic; thus, the results may not be generalizable to other centers with a different microbiological ecology. Finally, specific administered therapy, including corticosteroids and antibiotics, was not recorded. However, the main aim of the study was to report the epidemiology of both bacterial and fungal co-infections/superinfections in COVID-19 patients admitted to ICU and to investigate which CT imaging features may help in their detection.

## 5. Conclusions

There are still many open questions regarding the evolution of COVID-19, especially in critically ill patients admitted to ICU, in which it can be heterogeneous and unpredictable. The present study emphasizes the concern of bacterial and fungal infections among ICU patients hospitalized with COVID-19, which is a frequent complication. In particular, Gram-negative bacteria were confirmed as the most common co-pathogens in this subgroup of patients, even if fungal co-infections and superinfections are common. Differentiating viral from secondary bacterial and fungal pulmonary infections remains a challenge for both radiologists and clinicians. Imaging plays an important role in monitoring critically ill COVID-19 patients and in detecting any suspicious CT imaging features that may suggest further laboratory investigations and appropriate antimicrobial therapy. In particular, the presence of consolidations, cavitations, and bronchiectasis should be warning signs for radiologists, since they are associated with the presence of bacterial and/or fungal co-pathogens. Finally, co-infections and superinfections play a critical role in the negative outcome of COVID-19 patients admitted to ICU, favoring the progression to severe and fatal disease.

## Figures and Tables

**Table 1:** Demographic, clinical, and radiological characteristics of the study population and comparison between patients with and without co-infections/superinfections.

	Total (*n* = 95)	Patients with Co-Infections/Superinfections (*n* = 63)	Patients without Co-Infections/Superinfections (*n* = 32)	*p*
**Age** [mean (SD)]	64.2 (±10.5)	63.9 (±9.7)	64.8 (±12.0)	n.s.
**Sex** [*n* (%)]				n.s.
Male	71 (74.7%)	48 (76.2%)	23 (71.9%)	
Female	24 (25.3%)	15 (23.8%)	9 (28.1%)	
**BMI** [mean (SD)]	29.5 (±5.2)	29.3 (±5.3)	30.4 (±4.7)	n.s.
**Comorbidities** [*n* (%)]				
Diabetes	17 (17.9%)	13 (20.6%)	4 (12.5%)	n.s.
Recent Transplant	5 (5.3%)	5 (7.9%)	0 (0.0%)	n.s.
Lymphoproliferative disorders	10 (10.5%)	7 (11.1%)	3 (9.4%)	n.s.
**White blood cell (×10^9^/L)** [mean (SD)]	10.6 (±7.8)	10.3 (±6.0)	11.4 (±10.7)	n.s.
**Lymphocyte (×10^9^/L)** [mean (SD)]	2.0 (±6.9)	1.3 (±4.0)	3.4 (±10.4)	n.s.
**COVID-19 related complications** [*n* (%)]				
Barotrauma	14 (14.7%)	10 (15.9%)	4 (12.5%)	n.s.
Pulmonary thromboembolism	16 (16.8%)	11 (17.5%)	5 (15.6%)	n.s.
Encephalic complication	5 (5.3%)	3 (4.8%)	2 (6.3%)	n.s.
Abdominal complication	21 (22.1%)	16 (25.4%)	5 (15.6%)	n.s.
**Mortality** [*n* (%)]	41 (43.2%)	33 (52.4%)	8 (25%)	**0.016**
**CT imaging features** [*n* (%)]				
Ground-glass opacities	68 (71.6%)	48 (75.0%)	20 (64.5%)	n.s.
Crazy paving	66 (69.5%)	47 (73.4%)	19 (61.3%)	n.s.
Consolidations	82 (86.3%)	59 (93.7%)	23 (71.9%)	**0.009**
Cavitations	16 (16.8%)	15 (23.8%)	1 (3.1%)	**0.010**
Bronchiectasis	17 (17.9%)	16 (25.4%)	1 (3.1%)	**0.009**
Reticulations	29 (30.5%)	21 (32.8%)	8 (25.8%)	n.s
Bronchial distortions	31 (32.6%)	25 (39.1%)	6 (19.4%)	n.s.
Pleural effusion	30 (31.6%)	22 (34.4%)	8 (25.8%)	n.s.
Pericardial effusion	5 (5.3%)	3 (4.7%)	2 (6.5%)	n.s.
Lymph nodes (>10 mm)	19 (20.0%)	14 (21.9%)	5 (16.1%)	n.s.

SD: standard deviation; BMI: Body Mass Index; ICU: Intensive Care Unit; CT: Computed Tomography; n.s.: non-significant.

**Table 2:** Microbiology isolated by type of infection.

	*n* (%)
**Patients with co-infections/superinfections**	63 (66.3%)
Bacterial co-infections/superinfections	33 (52.4%)
Bacterial and fungal co-infections/superinfections	27 (42.9%)
Fungal co-infections/superinfections	3 (4.8%)
Mixed co-infections/superinfections	23 (36.5%)
**Identified microbes**	162
Bacteria	130 (80.2%)
Gram-negative	87 (66.9%)
Gram-positive	43 (33.1%)
Fungi	32 (19.8%)
**Patients with pulmonary co-infection/superinfection**	52 (82.5%)
**Identified respiratory microbes**	81
*Aspergillus* spp.	19 (23.5%)
*Klebsiella pneumoniae*	16 (19.7%)
*Pseudomonas aeruginosa*	8 (9.9%)
*Acinetobacter baumannii*	8 (9.9%)
*Staphylococcus aureus*	7 (8.6%)
*Candida* spp.	6 (7.4%)
*Escherichia coli*	4 (4.9%)
*Enterococcus faecium*	3 (3.7%)
*Stenotrophomonas maltophilia*	3 (3.7%)
*Citrobacter* spp.	2 (2.5%)
*Corynebacterium striatum*	2 (2.5%)
*Streptococcus pneumoniae*	1 (1.2%)
*Morganella morganii*	1 (1.2%)
*Serratia mascescens*	1 (1.2%)
**Patients with bloodstream co-infection/superinfection**	43 (68.2%)
**Identified bloodstream microbes**	64
*Enterococcus faecium*	11 (17.2%)
*Acinetobacter baumannii*	9 (14.1%)
*Pseudomonas aeruginosa*	8 (12.5%)
*Klebsiella Pneumoniae*	8 (12.5%)
*Staphylococcus epidermidis*	8 (12.5%)
*Staphylococcus aureus*	5 (7.8%)
*Candida* spp.	4 (6.2%)
*Escherichia coli*	4 (6.2%)
*Enterococcus faecalis*	3 (4.7%)
*Enterobacter* spp.	3 (4.7%)
*Serratia marcescens*	1 (1.6%)
**Patients with urinary tract co-infection/superinfection**	16 (25.4%)
**Identified urinary microbes**	17
*Acinetobacter baumannii*	4 (23.5%)
*Candida* spp.	3 (17.6%)
*Escherichia coli*	3 (17.6%)
*Enterococcus faecium*	3 (17.6%)
*Klebsiella pneumoniae*	2 (11.8%)
*Pseudomonas aeruginosa*	1 (5.9%)
*Corynebacterium striatum*	1 (5.9%)

**Table 3:** Comparison of imaging findings of patients with and without at least one pulmonary bacterial or fungal co-infection/superinfection and with and without invasive pulmonary aspergillosis.

	Pulmonary Co-Pathogen (*n* = 52)	No Pulmonary Co-Pathogen (*n* = 11)	*p*	Invasive Pulmonary Aspergillosis (*n* = 19)	No Invasive Pulmonary Aspergillosis (*n* = 33)	*p*
**CT imaging features** [*n* (%)]						
Consolidations	52 (100%)	8 (72.7%)	**0.004**	19 (100%)	33 (100%)	-
Cavitations	16 (30.8%)	0 (0.0%)	0.052	14 (73.7%)	2 (6.1%)	**<0.001**
Bronchiectasis	16 (30.8%)	0 (0.0%)	0.052	15 (78.9%)	1 (3.0%)	**<0.001**

**Table 4:** Comparison of imaging findings of COVID-19-related ARDS at baseline and during follow-up CT.

	Baseline CT (*n* = 95)	Follow-Up CT (*n* = 33)	*p*
**CT imaging features** [*n* (%)]			
Ground-glass opacities	68 (71.6%)	17 (51.5%)	n.s.
Crazy paving	66 (69.5%)	18 (54.5%)	n.s.
Consolidations	82 (86.3%)	29 (87.9%)	n.s.
Cavitations	16 (16.8%)	10 (30.3%)	n.s.
Bronchiectasis	17 (17.9%)	6 (18.2%)	n.s.
Reticulations	29 (30.5%)	21 (63.6%)	**0.001**
Bronchial distortions	31 (32.6%)	19 (57.6%)	**0.011**
Pleural effusion	30 (31.6%)	13 (39.4%)	n.s.
Pericardial effusion	5 (5.3%)	1 (3.0%)	n.s.
Lymph nodes (>10 mm)	19 (20.0%)	8 (24.2%)	n.s.

CT: Computed Tomography; n.s.: non-significant.

## Data Availability

The data presented in this study are available on request from the corresponding author.
